# Enzymatic Preparation of 2,5-Furandicarboxylic Acid (FDCA)—A Substitute of Terephthalic Acid—By the Joined Action of Three Fungal Enzymes

**DOI:** 10.3390/microorganisms6010005

**Published:** 2018-01-09

**Authors:** Alexander Karich, Sebastian B. Kleeberg, René Ullrich, Martin Hofrichter

**Affiliations:** 1 Department of Bio- and Environmental Sciences, Technische Universität Dresden—International Institute Zittau, 02763 Zittau, Germany; alexander.karich@tu-dresden.de (A.K.); rene.ullrich@tu-dresden.de (R.U.); 2 Natural and Environmental Sciences, University of Applied Sciences Zittau/Görlitz, 02763 Zittau, Germany; sbkleeberg@yahoo.de

**Keywords:** HMF, hydroxymethylfurfural, UPO, unspecific peroxygenase, aryl alcohol oxidase, galactose oxidase, *Agrocybe aegerita*, *Pleurotus ostreatus*, *Pleurotus eryngii*, *Bjerkandera adusta*

## Abstract

Enzymatic oxidation of 5-hydroxymethylfurfural (HMF) and its oxidized derivatives was studied using three fungal enzymes: wild-type aryl alcohol oxidase (AAO) from three fungal species, wild-type peroxygenase from *Agrocybe aegerita* (*Aae*UPO), and recombinant galactose oxidase (GAO). The effect of pH on different reaction steps was evaluated and apparent kinetic data (Michaelis-Menten constants, turnover numbers, specific constants) were calculated for different enzyme-substrate ratios and enzyme combinations. Finally, the target product, 2,5-furandicarboxylic acid (FDCA), was prepared in a multi-enzyme cascade reaction combining three fungal oxidoreductases at micro-scale. Furthermore, an oxidase-like reaction is proposed for heme-containing peroxidases, such as UPO, horseradish peroxidase, or catalase, causing the conversion of 5-formyl-2-furancarboxylic acid into FDCA in the absence of exogenous hydrogen peroxide.

## 1. Introduction

Plastics such as polyethylene terephthalates (PET) have become an integral part of human life, and the world’s annual consumption of such plastics has grown to about 40 million tons in 2014 and is forecasted to increase to over 70 million tons in 2020 [1,2]. Currently, the bulk portion of plastics is derived from fossil carbon sources [3]. An increasing usage of fossil raw materials will inevitably end in the exhaustion of the world’s capacity. Thus, renewable raw materials and new ways of production have to be developed and must be implemented in prospective industry. For some time, the fructose conversion product 5-hydroxymethylfurfural (HMF), a five-membered aromatic heterocycle, has come into the focus of polymer chemists as an alternative building block for the synthesis of PET-analogous polyesters deriving from renewable sources, such as starch [4,5].

In addition to bacterial whole-cell conversions [6,7], in the past years, some enzymatic reactions, including enzyme cascades, have been reported to produce FDCA from HMF by using flavin-dependent oxidoreductases, such as fungal aryl alcohol oxidase (AAO, EC 1.1.3.7) and heme-thiolate peroxidases (unspecific peroxygenase/UPO exclusively produced by fungi, EC 1.11.2.1; fungal chloroperoxidase/CPO, EC 1.11.1.10), or combinations of them [8,9,10,11]. Furthermore, in a recent patent, fungal galactose oxidase (GAO; EC 1.1.3.9), a rather unspecific copper-containing enzyme that oxidizes diverse sugars and alcohols [12], was reported to oxidize HMF in cooperation with UPO [8]. To our best knowledge, however, mixtures of AAO, GAO, and UPO have not been studied so far regarding HMF oxidation. Such an approach would not just simply combine different fungal enzymes that convert HMF, but also a peroxide-consuming biocatalyst with two peroxide-producing enzymes. Moreover, AAO, GAO, and UPO are glycosylated enzymes that are secreted by fungi into their microenvironments [13,14,15], and hence, they are rather robust when compared to intracellular or periplasmic bacterial enzymes [16]. Herein, we describe a new setup for a multi-enzyme cascade that converts HMF into FDCA, while using a combination of solely fungal oxidoreductases as catalytic system.

## 2. Materials and Methods 

### 2.1. Enzyme Preparations

Recombinant galactose oxidase (GAO) and catalase (Cat) expressed in *Aspergillus oryzae* were provided by Novozymes AS (Bagsværd, Denmark). Wild-type *Aae*UPO was produced and purified, as described previously [17]. The final *Aae*UPO preparation had a specific activity of 82 U mg^−1^ measured with veratryl alcohol as substrate according to Ullrich et al. [17]. Superoxide dismutase (SOD), glucose oxidase (GOD), and horseradish peroxidase (HRP) were purchased from Sigma-Aldrich (Schnelldorf, Germany).

Wild-type AAOs from *Pleurotus eryngii* (*Pery*AAO) and *Bjerkandera adusta* (*Badu*AAO) were produced in 10-L stirred-tank bioreactors (Biostat B+ Sartorius, Göttingen, Germany) containing 8 L of the culture media described below (the time courses of enzyme production are given in Figure S1 of the supplementary section). *Pleurotus ostreatus* (*Post*AAO) was cultivated in a 30-L stirred-tank bioreactor using 20 L culture medium (see Supplementary Material Section: Figure S2). The medium for *B. adusta* contained the following components: 3.0 g L^−1^ Na acetate, 0.5 g L^−1^ NH_4_ tartrate, 0.3 g L^−1^ yeast extract, 2.0 g L^−1^ K_2_HPO_4_, 0.5 g L^−1^ MgSO_4_·7 H_2_O, 0.1 g L^−1^ CaCl_2_, 0.01 g L^−1^ FeSO_4_·7 H_2_O; pH was adjusted to 5.5 with acetic acid. Three days after inoculation with 1 L of a liquid preculture (aeration 3.5–4.0 L min^−1^, 100 rpm), AAO production was stimulated by adding sterile-filtered veratryl alcohol (0.5 mM final concentration), and afterwards, cultivation was continued over seven days. For both *Pleurotus* species, the following medium was used to produce AAO: 10.0 g L^−1^ glucose, 1.0 g L^−1^ Na acetate, 2.0 g L^−1^ yeast extract, 5.0 g L^−1^ peptone (from soybeans), 2.0 g L^−1^ KH_2_PO_4_, 0.5 g L^−1^ MgSO_4_·7 H_2_O, 0.1 g L^−1^ CaCl_2_, 0.01 g L^−1^ FeSO_4_·7 H_2_O; pH was adjusted to 5.2 with HCl. Veratryl alcohol was not supplemented and cultivation was carried out over seven days.

AAO-containing culture liquids (crude extracts) were filtered through cotton fabric and then, the filtrates were frozen and thawed to remove dissolved oligosaccharides by precipitation. Afterwards, the liquid was centrifuged (Sorvall Lynx 6000; Fiberlite™ F9-6 x 1000 LEX, 9000 rpm, Thermo Scientific™, Schwerte, Germany) and filtered through glass microfiber filters (GF/F 125 mm; GE healthcare LS Whatman™, Dornstadt, Germany). The particle-free filtrates were concentrated with an ultra-tangential filtration system using a 10-kDa cut-off membrane (Sartocon Slice Disposable; 3061463901E-SW; Sartorius stedim, Goettingen, Germany). Partial AAO purification was achieved by two steps of ion exchange chromatography steps using Q-sepharose^®^ (26 mm × 200 mm) and Mono Q^®^ columns (10 mm × 100 mm; both GE Healthcare, Dornstadt, Germany). Eventually, size exclusion chromatography (SEC) was applied using a Sephadex^®^ 75 column (GE Healthcare, Dornstadt, Germany). More detailed information, including FPLC elution profiles and an exemplary purification table, are given in the Supplementary Material Section (Figures S3–S7 and Table S1). AAO activity was measured, as described in Kirk and Farrell [18]. Final specific activities of *Badu*AAO, *Pery*AAO, and *Post*AAO were 28.1 U mg^−1^, 39.0 U mg^−1^ and 80.6 U mg^−1^, respectively.

### 2.2. Chemicals

Hydroxymethylfurfural (HMF), 2,5-diformylfuran (DFF), 5-formyl-2-furancarboxylic acid (FFCA), 5-hydroxymethyl-2-furancarboxylic acid (HMFCA), and 2,5-furandicarboxylic acid (FDCA) were purchased from Sigma-Aldrich (Schnelldorf, Germany) with the highest purity grade available. All of the other chemicals used (including culture media components) were obtained from VWR International (Dresden, Germany).

### 2.3. HPLC Analyses

HMF and its oxidation products were analyzed using an Agilent 1200 series HPLC system (Agilent, Waldbronn, Germany) equipped with a diode array detector operating between 210 and 500 nm, as well as at specific wavelengths (254, 270, 280, and 285 nm) for calibration. Separation of analytes occurred on a Resex column (ROA, organic acid H^+^ 8%; Phenomenex, Aschaffenburg, Germany) at 50 °C and the liquid phase was 0.05 N H_2_SO_4_ (isocratic conditions, flow rate 0.75 mL min^−1^). An exemplary HPLC elution profile is shown in Figure S8.

### 2.4. Reaction Setups

The reaction setup to evaluate pH dependencies of oxidoreductases in the oxidation of HMF and its derivatives was set as follows: 2 mM substrate, 50 mM phosphate buffer of varying pH (3.0 to 9.0), instant one-time-addition of 1 mM H_2_O_2_ in the case of *Aae*UPO with HMF and DFF as substrates or slow supply of 2 mM H_2_O_2_ via a syringe pump over two hours with FFCA and HMFCA as substrates; the total volume was 500 µL in 1.5-mL HPLC vials in all of the cases. The applied enzyme concentrations were 0.6 mg mL^−1^, 0.06 mg mL^−1^ and 0.012 mg mL^−1^ of GAO, AAOs, and *Aae*UPO, respectively; in the case of FFCA oxidation by *Aae*UPO, only 0.0012 mg mL^−1^ were used. The reaction mixtures were stirred with a magnet (*Aae*UPO) or constantly shaken (oxidases) for two hours (or for 15 min during HMF and DFF conversion by *Aae*UPO) and stopped by adding 50 µL trichloroacetic acid (50%) or by heating (95 °C) for 3 min in the case of FFCA samples.

The reaction setup for the determination of apparent kinetic constants (at varying substrate concentrations) contained in a total volume of 500 µL:50 mM potassium phosphate buffer (KP_i_, pH 6.0), 0.5 to 140 mM substrate (HMF or DFF), as well as 4 µM, 2 µM, and 0.111 µM of GAO, AAOs, and *Aae*UPO, respectively. Reactions were stopped with 50 µL sodium azide (10 mM).

The oxidation of FFCA by selected enzymes was studied in more detail in separate experiments. Reaction mixtures with AAOs contained in a final volume of 0.5 mL following components: 2.5 mM FFCA, 50 mM KP_i_ (pH 6, 7, or 7.5), 0.02 mg mL^−1^ AAO (*Pery*AAO, *Post*AAO, or *Badu*AAO), as well as optionally 2.2 µg mL^−1^ catalase (Cat). They were shaken at 175 rpm for up to 76 h at room temperature. Reactions with *Aae*UPO were analogously carried out (2.5 mM FFCA, 50 mM KP_i_, pH 7.25) and contained diverse combinations of *Aae*UPO (0.04 mg mL^−1^), Cat (2.2 µg mL^−1^), SOD (10 µg mL^−1^), HRP (1 mg mL^−1^) and H_2_O_2_ (5 mM). Furthermore, FFCA (2.5 mM) was treated with *Aae*UPO (0.04 mg mL^−1^) in combination with GOD (0.1 mg mL^−1^), glucose (50 mM), and Cat (2.2 µg mL^−1^); the latter reaction was also performed in the absence of *Aae*UPO. Further controls of the aforementioned reaction setups contained: 2.5 mM FFCA in 50 mM KP_i_ (pH 7.25) and optionally 10 mM H_2_O_2_, as well as heat-inactivated *Aae*UPO (0.12 and 0.012 mg mL^−1^ corresponding to formerly 10 and 1 U mL^−1^, respectively) or 50 mg mL^−1^ of porcine hemoglobin or crude hemin (5 mg mL^−1^) or 1 mM sodium azide.

FFCA oxidation by AAOs was followed at three pH values and the respective reaction mixtures comprised of 50 mM KP_i_ (pH 6.0 or 7.5), 6 µg mL^−1^ of *Badu*AAO, *Post*AAO or *Pery*AAO, and 5 mM FFCA. To evaluate whether FFCA oxidation catalyzed by AAOs can be inhibited by H_2_O_2_, FFCA was incubated for 24 h (25 °C, 175 rpm) with *Badu*AAO, *Post*AAO, and *Pery*AAO in the presence of different concentrations of H_2_O_2_ (0 to 20 mM).

Eventually, a model cascade reaction combining GAO (0.6 mg mL^−1^), *Pery*AAO (0.32 mg mL^−1^), and *Aae*UPO (0.098 mg mL^−1^) was realized in a larger reaction volume (10 mL) containing 10 mM HMF as substrate (50 mH KP_i_, pH 6.0). Samples of 50 µL were taken every 40 min from this reaction solution and analyzed by HPLC. Exogenous H_2_O_2_ was not added and *Aae*UPO was fully supplied with peroxide through the oxidase reactions. All of the enzymatic measurements were carried out in triplicate; if not otherwise indicated, standard deviations were <5%.

## 3. Results

### 3.1. Oxidation of HMF and Its Derivatives

**Figure 1:**
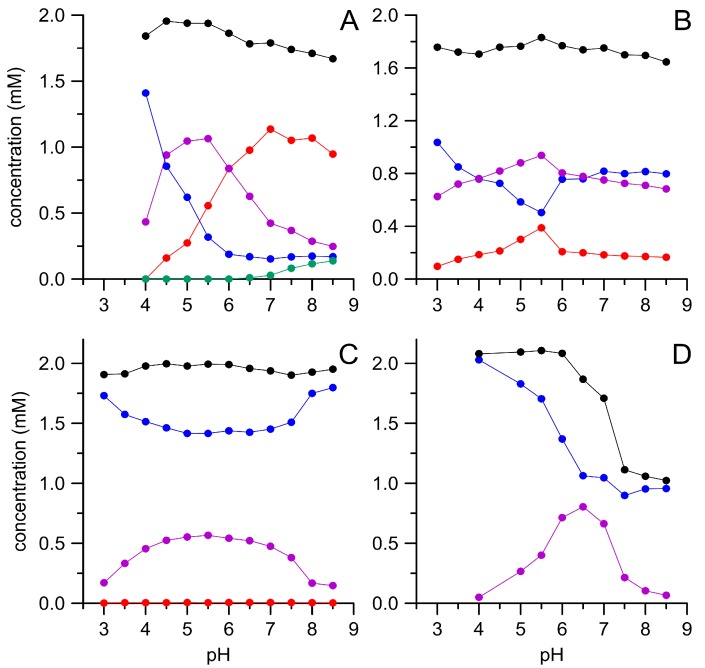
Enzymatic oxidation of 5-hydroxymethylfurfural (HMF) by different oxidases in dependence of the pH. *Pery*AAO (**A**), *Post*AAO (**B**), *Badu*AAO (**C**), and galactose oxidase (GAO) (**D**); HMF (blue), 5-diformylfuran (DFF) (violet), HMFCA (green), 5-formyl-2-furancarboxylic acid (FFCA) (red), and sum of HMF and its derivatives (black). Data points are means of triplicate measurements with standard deviations <5%.

All four oxidases tested were able to convert substantial amounts of HMF, which became evident by the concomitant decrease of HMF and the appearance of DFF and other oxidation products (Figure 1). However, the individual enzymes noticeably differed regarding their pH dependencies and product patterns. Thus, both *Pleurotus* AAOs further oxidized the primary product DFF under the formation of FFCA. This was more pronounced with *Pery*AAO at pH ≥ 7.0 and even at pH 8.5 still over 50% of the applied HMF was transformed into FFCA. In addition to the latter compound, also small amounts of HMFCA were detectable under these conditions. Since HMFCA did not serve as substrate for *Pery*AAO, this implies that the enzyme oxidizes HMF—below pH 7.0—via DFF to FFCA, while above this value, the oxidation can proceed via DFF, but may also lead to HMFCA that is a dead-end product. In contrast, *Post*AAO oxidized HMF exclusively via DFF in a broad pH range (3.0–8.5) and did not form HMFCA. *Badu*AAO was not efficient in oxidizing HMF and formed only trace amounts of FFCA (<0.1 mM). On the other hand, it was active in a broad pH range (3.0–8.5; similar as *Post*AAO). GAO worked best at pH 6.5, but did not produce FFCA. It was the only of the tested enzymes, which was capable of oxidizing HMFCA to FFCA. Interestingly, the sum of HMF and its oxidation products in the reaction mixture dropped to about 1 mM (Figure 1D, black dots) when GAO faced DFF above pH 6.0, indicating the formation of further unidentified products.

**Figure 2:**
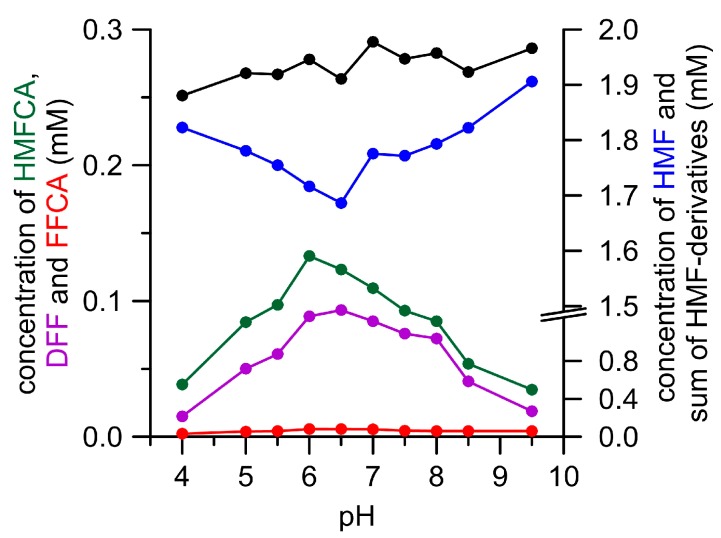
pH-Dependency of HMF oxidation by *Aae*UPO. HMF (blue), DFF (violet), HMFCA (green), FFCA (red), and sum of HMF derivatives (black). Only traces of 2,5-furandicarboxylic acid (FDCA) (<0.01 mM) were formed under these conditions.

The pH dependency of enzymatic DFF oxidation was studied in a separate experiment with *Pleurotus* AAOs and GAO. The former caused merely the formation of small amounts of FFCA and did not develop a distinctive pH optimum for DFF oxidation. GAO did not oxidize DFF into FFCA at all, but produced other unknown products above pH 6.0 (see supplementary data Figures S9 and S10). *Aae*UPO oxidized HMF to DFF and HMFCA at an almost stable and pH-independent ratio (1:1.4; Figure 2). The highest amounts of both the products were formed at pH 6 and 6.5. The optimum for DFF oxidation by *Aae*UPO was found to range between pH 6.5 and 7.0, and thus occurred in the same range as HMF oxidation (see supplementary data Figure S11). Concluding from all of these tests, a neutral pH seems to be most suitable to oxidize HMF and DFF with oxidases and peroxygenase.

Apparent catalytic constants for enzymatic HMF and DFF oxidation are given in Table 1; the corresponding Michaelis-Menten plots are shown in the supplementary data section (Figures S12 and S13). The extent of DFF-to-FFCA conversion by AAOs was not sufficient to calculate catalytic constants, however, it was possible to estimate specific activities, which amounted to 0.3 U mg^−1^ and 0.18 U mg^−1^ for *Post*AAO and *Pery*AAO, respectively. On this basis, we concluded that the reaction (DFF → FFCA) proceeds roughly 500 times slower than the oxidation of the AAO assay substrate veratryl alcohol. Interestingly, the specific constants (‘catalytic efficiencies’, *k*_cat_/*K*_M_) of *Aae*UPO for HMF and DFF were almost identical (36.6 × 10^3^ and 35.6 × 10^3^ M^−1^ s^−1^), although the respective turnover numbers (*k*_cat_, 13,300 and 1750 min^−1^) and Michaelis-Menten constants (*K*_M_, 607 and 82 µM) differed by an order of magnitude.

### 3.2. FFCA Oxidation

**Figure 3:**
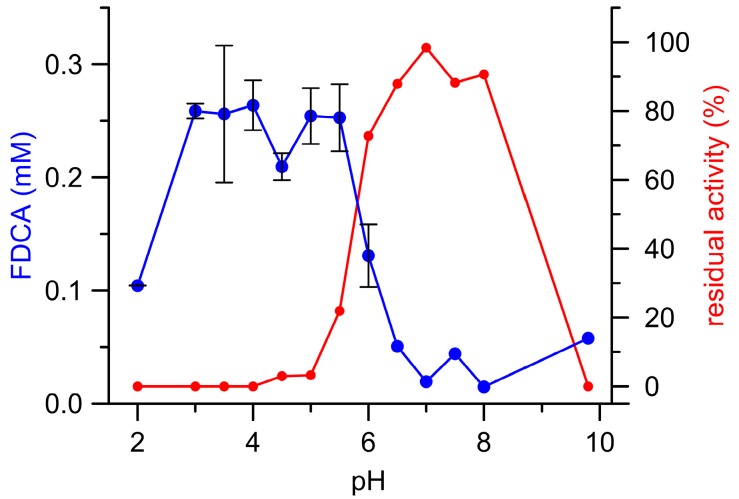
*Aae*UPO catalyzed formation of FDCA (blue) from FFCA as well as the corresponding residual UPO activities (red) in dependence on the pH.

Figure 3 illustrates the pH dependency of (final) FFCA conversion to FDCA catalyzed by *Aae*UPO and the corresponding residual enzymatic activity after the reaction (‘enzyme survival’). Interestingly, most FDCA was formed below pH 6.0 where a substantial loss of UPO activity was observed (complete enzyme inactivation between pH 2.0 and 4.0; in other words, the enzyme oxidized the substrate on the expense of its ‘health’/functionality). The resulting total turnover number (ttn) was 170, which is rather unfavorable for an enzymatic conversion. In contrast, UPO activity above pH 6.0 (pH 6.5–8.5) was almost completely preserved, albeit the amounts of FDCA formed were more than five times lower when compared to acidic conditions. Consequentially, pH 6.0 turned out to be the most suitable pH with regard both to the amount of FDCA formed and UPO’s process stability.

Repeated analyses (after 24 and 48 h) of *Aae*UPO-containing samples (and controls) revealed a continued FDCA production, although H_2_O_2_ had already depleted (data not shown). This phenomenon needed closer inspection and Table 2 summarizes the respective findings. FDCA (0.3 mM) appeared also in the reaction mixture when FFCA was incubated with *Aae*UPO in the absence of exogenous H_2_O_2_ (or a peroxide-generating enzyme) and the addition of H_2_O_2_ increased the amount to just 0.4 mM. H_2_O_2_ alone oxidized FFCA merely to a negligible extent. When *Aae*UPO was combined with Cat (that actually decomposes H_2_O_2_), FDCA formation (1.9 mM) was still intensified, while SOD did neither stimulate nor affect the reaction. Interestingly, the heme enzymes Cat and HRP caused substantial FFCA oxidation (1.4 and 0.5 mM FDCA formation, respectively, which was in fact higher than that caused by *Aae*UPO (note, however, that the enzyme amounts of Cat/HRP and *Aae*UPO varied by (several) orders of magnitude)). In contrast, the flavin enzyme GOD (actually producing peroxide through glucose oxidation) inhibited FFCA oxidation by Cat, as well as by *Aae*UPO and Cat (Table 2). Control reactions with *Aae*UPO inactivated by heat or sodium azide, inactivated Cat, hemoglobin, or crude hemin did not provoke FDCA formation.

Although AAOs did not produce FDCA when the reaction started from HMF, it is worth mentioning that all three AAOs tested did produce FDCA when FFCA was supplied as substrate over reaction times of 24 h and more (compare supplementary data Table S2). However, when varying the concentrations of H_2_O_2_ were supplemented to the reaction mixtures, FDCA formation decreased (see supplementary data Figure S14). Thus, *Badu*AAO and *Post*AAO produced 0.3 and 0.2 mM FDCA, respectively, in the absence of H_2_O_2_, but no FDCA when 0.25 mM peroxide was present. That means, the final oxidation of FFCA to FDCA by *Post*AAO will be always completely inhibited by H_2_O_2_ formed during initial HMF oxidation (or in other words, the sensitivity of the final reaction for H_2_O_2_ is about 300-times higher than that of the initial reaction). In contrast, *Pery*AAO produced from the outset twice as much FDCA (0.5 mM), and this was not affected by moderate amounts of H_2_O_2_ (0.1–7.5 mM; FDCA formation even continued—albeit to smaller extent—above 10 mM H_2_O_2_; Figure S14).

### 3.3. Combined Cascade Reaction

**Figure 4:**
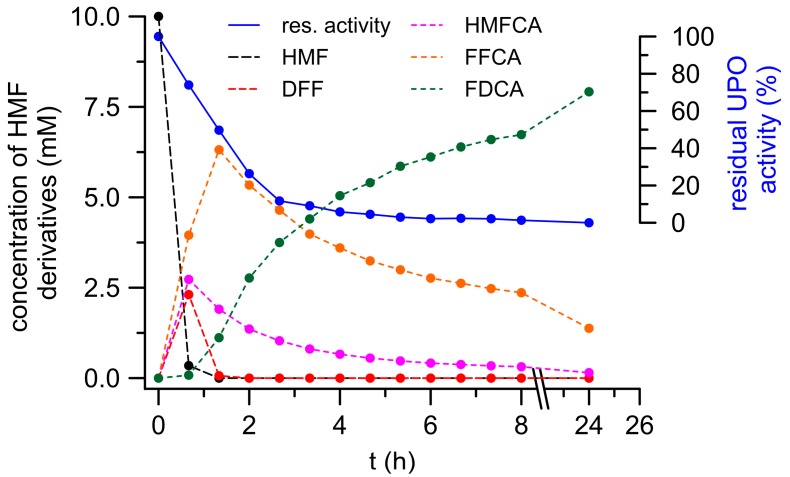
FDCA formation in a cascade-reaction of three oxidoreductases (GAO, *Pery*AAO, and *Aae*UPO).

Figure 4 shows the time-dependent formation of HMF oxidation products catalyzed by a cocktail of *Pery*AAO, GAO and *Aae*UPO. The reaction setup was chosen based on the results presented above. UPO was included for two reasons: to utilize H_2_O_2_ produced by oxidases and to oxidize HMF and its oxidized derivatives, including FFCA. GAO should oxidize HMF and particularly HMFCA produced by UPO (while forming H_2_O_2_), and *Pery*AAO may efficiently oxidize HMF to DFF, along with substantial H_2_O_2_ production (to be used by UPO). Under such conditions, more than 95% of the applied HMF (9.7 mM) was already converted after 45 min (first sampling) and after 75 min, it was fully consumed. Concomitantly, DFF and HMFCA appeared in the reaction mixture reaching their maximum concentrations of 2.4 mM and 2.7 mM, respectively, already after 45 min. DFF rapidly disappeared within the next 40 min, whereas HMFCA was rather slowly converted (about 50% within the next 75 min). In both cases, further oxidation yielded FFCA that reached its maximum of 6.3 mM after 75 min (which corresponds to 70% conversion of the applied HMF). Final conversion of FFCA to FDCA turned out to be the bottleneck of the overall oxidation and lasted for the complete remaining reaction time. Nevertheless, FDCA constantly increased and finally reached a concentration of 7.9 mM, which corresponds to an 80% yield related to HMF applied (a total of 15.5 mg FDCA). Furthermore, after 24 h reaction time, the total amount of HMF oxidation products was 9.5 mM, which means an almost complete mass balance.

## 4. Discussion

**Figure 5:**
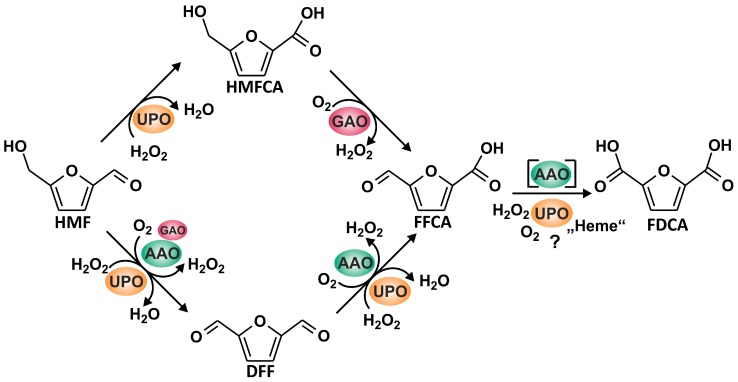
Reaction sequence leading from HMF to FDCA realized by the joined action of AAO, GAO, and *Aae*UPO.

We have tested five different fungal oxidoreductases (three AAOs, recombinant GAO, and *Aae*UPO) for their ability to oxidize HMF and its oxidized derivatives. A summarizing formula scheme illustrating the possible reactions is given in Figure 5. The three AAOs tested were fungal wild-type proteins produced by their natural hosts (homologous expression). To our best knowledge, only recombinant AAOs have been used so far in studies dealing with HMF oxidation [7]. On the other hand, *Pleurotus ostreatus* was reported to detoxify HMF (that can harm microbes and inhibit fungal cellulolytic enzymes) by means of AAO and it was proposed to integrate the respective gene into fermentation organisms, such as *Saccharomyces cerevisiae* [19].

All of the tested oxidases exhibited pH optima for HMF oxidation around pH 6.0. This corresponds well with earlier findings using recombinant AAO and a mutant GAO [10,20]. Although the product spectrum of the two *Pleurotus* AAOs was rather similar (with the exception of FDCA formation by *Pery*AAO at alkaline pH), their individual pH dependencies were notwithstanding fairly different. Carro et al. [10] proposed that FFCA is formed from HMF without the intermediate (DFF) leaving the active site. This interesting assumption is supported by some of our data, especially by the weak direct DFF oxidation when it was applied as sole substrate (low specific activities <0.3 mU mg^−1^) and the fact that neither *Pery*AAO nor *Post*AAO developed a distinct pH optimum for direct DFF oxidation. However, in the course of our pH dependency studies with HMF, both *Pleurotus* AAOs formed reasonable amounts of free DFF (about 1 mM at pH 5.5) that obviously had left the active site to be further oxidized after re-entering the active site (compare Figure 1). Maybe, only part of the DFF formed stayed in the active center, while another fraction diffused into the medium. Based on our findings, we assume that the molecular properties of *Pery*AAO’s and *Post*AAO’s active sites—despite high sequence homology (95%)—differ substantially [21,22]. The molecular architecture of the latter may allow adjusting an appropriate pH in the nano-environment of its active site, minimizing the pH influence of the surrounding reaction solvent. *Pery*AAO, on the other hand, is seemingly lacking such a pH-stabilizing mechanism, and hence more affected by the pH of the reaction solvent. These assumptions could be assessed in future studies by comparing the enzymes’ crystal structures or reliable homology models [22,23]. 

When comparing the catalytic constants of wild-type AAOs (especially those of *Pery*AAO; Table 1) with the data published by Carro et al. [10] for recombinant *Per*yAAO, noticeable differences in *k*_cat_ and *K*_M_ become evident. The *k*_cat_ value for HMF of the wild-type enzyme we used is about ten times higher and the *K*_M_ value 20 times lower than the values of recombinant AAO [10]. This can be explained in two ways: first, the lacking glycosylation of recombinant AAO (expressed in *E. coli*) may have affected the enzymatic performance [10,24], or second, the different methods used for determining the apparent catalytic data, i.e., direct vs. indirect measurements, influenced the calculation of constants. *Aae*UPO exhibited the highest specific constants (*k*_cat_/*K*_M_) both for HMF (3.66 × 10^4^ M^−1^ s^−1^) and DFF (3.56 × 10^4^ M^−1^ s^−1^), indicating a certain potential of this enzyme type for further catalytic improvement. Interstingly, *Aae*UPO’s *k*_cat_/*K*_M_ values for HMF and DFF are in the same range as that of the model substrate veratryl alcohol (3.58 × 10^4^ M^−1^ s^−1^) [17]. Hence, we can assume that *Aae*UPO has significantly contributed to the HMF and DFF oxidation in the cascade experiment and worked jointly with AAOs and GAO.

GAO turned out to have an exceptional low specific constant for HMF oxidation (4.9 M^−1^ s^−1^). At first glance, this appears astonishing when considering that GAO effectively oxidizes diverse monosaccharides such as galactose and glucose and hence, should be able to oxidize sugar derivatives like HMF as well [25]. However, despite its formal similarity to furanoid sugars, HMF represents—in accordance to Hückel’s rule—an aromatic system (because one of the lone pairs of electrons on the oxygen atom is delocalized into the ring, creating a ‘4*n* + 2’ system). Considering that GAO also oxidized HMFCA, resulting in FFCA and H_2_O_2_ formation, this nevertheless makes the enzyme an interesting candidate for HMF cascade reactions. Thus, HMFCA produced by UPO (that is not a substrate for the other cascade enzymes) can be further used and stoichiometrically returned into the reaction. Moreover, it might be advantageous that HMFCA acts as an ‘H_2_O_2_-sink’ to keep the stationary H_2_O_2_ concentration in the reaction mixture at a moderate level and prevent UPO from inactivation [26].

The fact that AAOs did not produce FDCA when HMF was supplied as substrate, but did produce it when FFCA was applied as the sole substrate can be explained by a sort of end-product inhibition. Thus, the two stoichiometric equivalents of H_2_O_2_ formed during the oxidation of HMF and DFF by AAO may inhibit the oxidation of FFCA to FDCA. A strong indication for the correctness of this assumption is given in Figure S14. However, we cannot explain, for which reason the AAOs were affected by varying H_2_O_2_ concentrations in such different ways. Possibly, the differences in stability towards H_2_O_2_ are related to the generally differing pH dependencies (compare Figure 1 and Figure S14). As far as we know, the substantial inhibition of AAO by H_2_O_2_ has not been reported yet.

Production of FDCA was also achieved with *Aae*UPO, even if the amount of FDCA that was formed was insufficient when FFCA was applied as sole substrate and H_2_O_2_ supplied via syringe pump (compare Figure 3). *Aae*UPO’s inactivation at pH below 6.0 may have been caused by excess H_2_O_2_ via compound III formation and heme bleaching, which was already observed for this enzyme in a previous study [26]. In contrast, above pH 6.0, *Aae*UPO’s intrinsic catalase activity may have taken effect and decomposed some H_2_O_2_ so that the enzyme stability increased (but at the expense of FFCA oxidation that competed with the catalase activity) [26]. This finding partly contrasts previous results where *Aae*UPO was added to a reaction mixture, which contained afore-produced H_2_O_2_ (6 mM by AAO) and residual FFCA (3 mM) that was oxidized to FDCA over 120 h without additional supplementation of H_2_O_2_ [10]. Closer inspection of this fact (compare Table 2) surprisingly demonstrated that *Aae*UPO is capable of forming FDCA from FFCA in the absence of its natural co-substrate H_2_O_2_. The same phenomenon was observed for Cat (a supposedly highly specific heme enzyme that actually decomposes H_2_O_2_ [27]; compare Table 2). This implies that auto-catalytically formed peroxide, the formation of which had been shown for Mn peroxidase [28], was not responsible for UPO-catalyzed FFCA oxidation. Similar applies to SOD; when it was present in the reaction mixture, along with *Aae*UPO and/or Cat, the product yields did not significantly change, i.e., superoxide (O_2_^•−^) was not relevant for the reaction catalyzed by UPO (nor UPO/Cat) [28,29]. However, the decrease of FDCA formation in the presence of GOD (along with *Aae*UPO and/or Cat) led us conclude that dioxygen (O_2_) may be involved in the reaction.

Anyway, the H_2_O_2_-independent formation of FDCA catalyzed by *Aae*UPO, Cat or HRP must be the result of a true enzymatic oxidation process, since it was azide-sensitive and was not brought about by plain hemin or hemoglobin. FDCA formation by *Aae*UPO or Cat was fully inhibited when sodium azide (NaN_3_) was added, which is known to inactivate heme proteins via catalytic formation of *meso*-azidoprotoporphyrin IX and/or oxidation of the apoprotein [30]. When considering all these results, it is plausible that *Aae*UPO (HRP, Cat) possess an oxidase-like activity towards FFCA. This activity was most pronounced in *Aae*UPO that converted FFCA at much lower concentration (~100-fold) than Cat or HRP. Oxidase-like activities have already been reported for some other heme-containing peroxidases, including HRP (well-studied oxidation of indole acetic acid) and a β,β-carotene-cleaving fungal peroxidase of the DyP-type [29,31,32,33], but the underlying mechanisms are not fully understood yet. In consequence, we propose that the FDCA formation observed by Carro et al. [10] may be not attributed to *Aae*UPO’s “usual” peroxygenating activity, but to a putative oxidase-like activity. Another FDCA-producing cascade reaction described in the literature combined a periplasmatic bacterial aldehyde oxidase, HRP, GAO, and Cat [9,34]. However, the amounts of Cat and HRP that were used in this reaction were markedly high (0.3 mg mL^−1^ and 0.2 mg mL^−1^, respectively) and it was not mentioned whether controls solely with catalase were run and if so, whether they produced FDCA. 

Overall, our results demonstrate that it is possible to establish enzyme cascade reactions that combine fungal UPO with different peroxide-generating fungal oxidases, such as AAO and GAO, which were never used in combination in previous studies, to produce substantial amounts of FDCA starting from HMF (compare Figure 5). However, the amount of applied UPO to obtain sufficient amounts of FDCA is still far from being realistic (at the moment about 50 U protein for one mg product), but it may be the starting point for the further development of enzyme-based conversion processes. Although a product yield of 80% along with a mass balance of 95% is a reliable basis for further studies [9,35,36], the setup that we propose will need comprehensive improvement and optimization of the reaction, including minimized oxidase usage to keep the local H_2_O_2_ concentration at levels that do not damage UPO [26]. Further process development should focus on the proposed reaction scheme given in Figure 5. *Aae*UPO activity was found to decrease mainly in the beginning of the reaction when HMF and DFF (as suitable oxidase substrates) are being converted. With a delay of about 60 min, the decrease of *Aae*UPO activity slowed down, precisely at that time when only HMFCA, FFCA, and FDCA were left in the reaction mixture. Eventually, it should be mentioned that such an enzyme-based process development will also face various technical challenges to be met in order to achieve a reliable performance. For example, the activity of oxidases is boosted, if pure dioxygen will be used instead of air or if the solubility of dioxygen in the liquid phase will be increased by applying elevated pressure [37].

## Figures and Tables

**Table 1:** Apparent catalytic constants (Michaelis-Menten constant, turnover number, specific constant) of three fungal oxidases (AAO, GAO) and an unspecific peroxygenase (UPO) for HMF and DFF oxidation at pH 6.0.

Enzyme	Substrate	*K*_M_ (mM)	*k*_cat_ (min^−1^)	*k*_cat_/*K*_M_ (M^−1^ s^−1^)
*Aae*UPO	HMF	6.07	13,333	36,610
	DFF	0.82	1752	35,622
*Pery*AAO	HMF	36.3	219	100
*Post*AAO	HMF	7.2	177	411
GAO	HMF	142	42	4.9

**Table 2:** FDCA formation from FFCA (2.5 mM) in the presence of different enzymes, enzyme cocktails and effectors. Reactions were performed in phosphate buffer (50 mM, pH 7.25) over 48 h. Concentrations given are mean values of three measurements with standard deviation.

Reaction Setup	FDCA (mM)
*Aae*UPO	0.31 ± 0.01
*Aae*UPO/H_2_O_2_	0.40 ± 0.05
H_2_O_2_	0.05 ± 0.01
Hemoglobin/H_2_O_2_	0.06 ± 0.00
Hemin/H_2_O_2_	0.06 ± 0.00
*Aae*UPO/Cat	1.88 ± 0.11
*Aae*UPO/SOD	0.33 ± 0.02
*Aae*UPO/Cat/SOD	1.98 ± 0.09
*Aae*UPO/Cat/SOD/H_2_O_2_	2.08 ± 0.07
Cat	1.39 ± 0.09
Cat/SOD	1.37 ± 0.08
*Aae*UPO/Cat/GOD	0.61 ± 0.03
Cat/GOD	0.08 ± 0.01
HRP	0.52 ± 0.01
UPO/sodium azide	0.00 ± 0.00
UPO (boiled)	0.00 ± 0.00
Cat/sodium azide	0.00 ± 0.00

## Supplementary Materials

The following are available online at www.mdpi.com/2076-2607/6/1/5/s1.

Supplementary File 1
