# Supplementary material for: Enzymatic Preparation of 2,5-Furandicarboxylic Acid (FDCA)—A Substitute of Terephthalic Acid—By the Joined Action of Three Fungal Enzymes
Source: Microorganisms. 2018 Jan 9;6(1):5. doi: 10.3390/microorganisms6010005 (PMC5874619; doi:10.3390/microorganisms6010005)
Supplement: Supplementary File 1 [file microorganisms-06-00005-s001.pdf]

Supplementary material

1.1 Enzyme production

AAO of *Pleurotus ostreatus*
(30-L stirred tank)

- Medium: 10.0 g glucose (Merck); 1.0 g Na acetate (Merck); 2.0 g yeast extract (Merck); 5.0 g peptone (from soybeans, Merck); 2.0 g KH_2PO_4 (Merck); 0.5 g $MgSO_4$ x 7 H_2O (Merck); 0.1 g $CaCl_2$ (Merck); 0.01 g $FeSO_4$ x 7 H_2O (Merck); pH 5.2
- Aryl alcohol oxidase (AAO, E.C. 1.1.3.7): oxidation of veratryl alcohol to veratraldehyde at pH 5.0 (Kirk et al., 1986); 310 nm (ε = 9,300 $M^{-1}cm^{-1}$); 50 mM Na citrate (pH 5.0 or 5.5); 5 mM veratryl alcohol

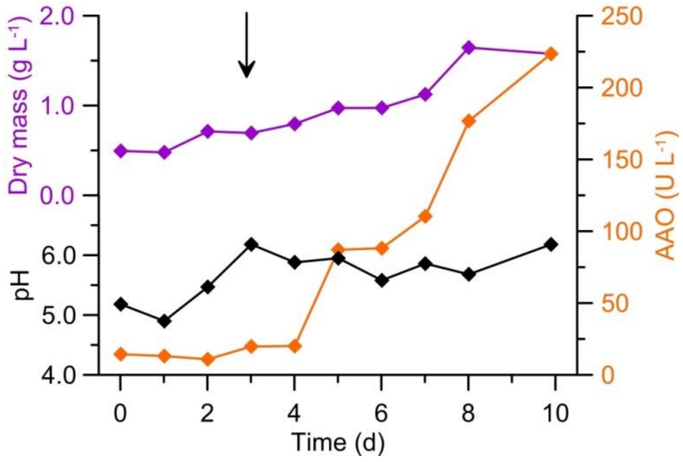

Fig S1: Time course of AAO production by *Bjerkandera adusta* in a stirred-tank bioreactor (with 8 L culture medium); the arrow indicates supplementation with veratryl alcohol to stimulate AAO production. Purple curve - dry mass; black curve – pH; orange curve - *Post*AAO activity

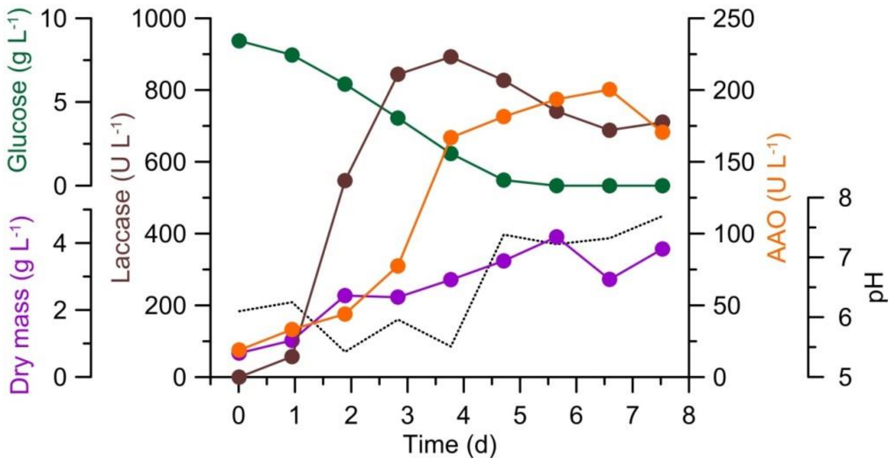

Fig. S2: Time course of AAO production by *Pleurotus ostreatus* in a 30-L stirred tank reactor (20 L medium). Purple curve - dry mass; green curve – glucose concentration; black curve – pH; orange curve - *Post*AAO activity; brown curve – Laccase Activity

1.2 Enzyme purification

Ion-exchange chromatography:
Sample preparation: dilution with 10 mM Na acetate pH 6.0 (resulting in a pH around 7)
Column: **Q-sepharose®**, 26 mm x 200 mm, GE Healthcare
Loading buffer: 10 mM Na acetate pH 6.0
Elution buffer: 10 mM Na acetate pH 5.7 + 2 M NaCl
Flow: 13 mL min^{-1}, loading with sample pump; washing with 1.5 CV; 50% B within 10 CV; fraction size 7 mL; around 2,700 U *Post*AAO were applied per run → recovery 2,800 U

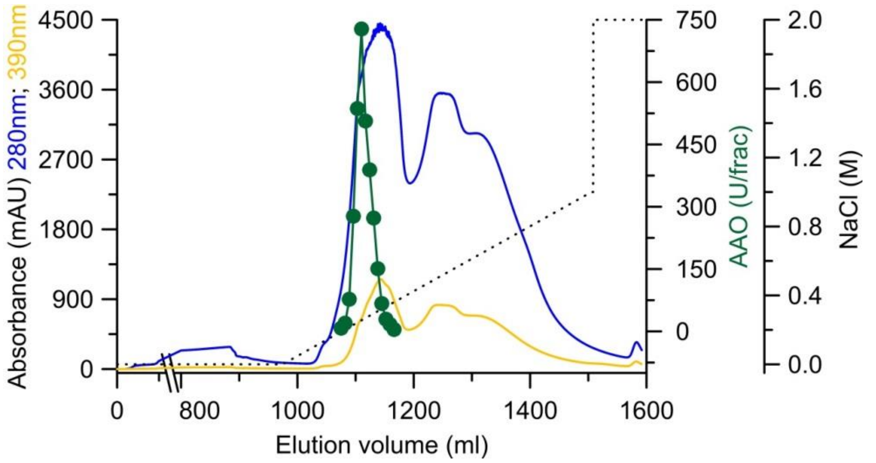

Fig. S3: Elution profile of *Post*AAO; anion exchange chromatography occurred on a Q-sepharose® column (26/200) after loading 2,700 U *Post*AAO.

Sample preparation: dilution & purging with 10 mM Na acetate (pH 6) using viva spins: 10 kDa cut-off
Column: **Mono Q®**, 10 mm x 100 mm, GE Healthcare
Loading buffer: 10 mM Na acetate pH 5.25
Elution buffer: 10 mM Na acetate pH 5.7 + 2 M NaCl
Flow: 6 mL min^{-1}, loading with sample pump; washing with 2.0 CV; 30% B within 15 CV; fraction size 2 mL; around 2,800 U *Post*AAO were applied per run → recovery 2,600 U

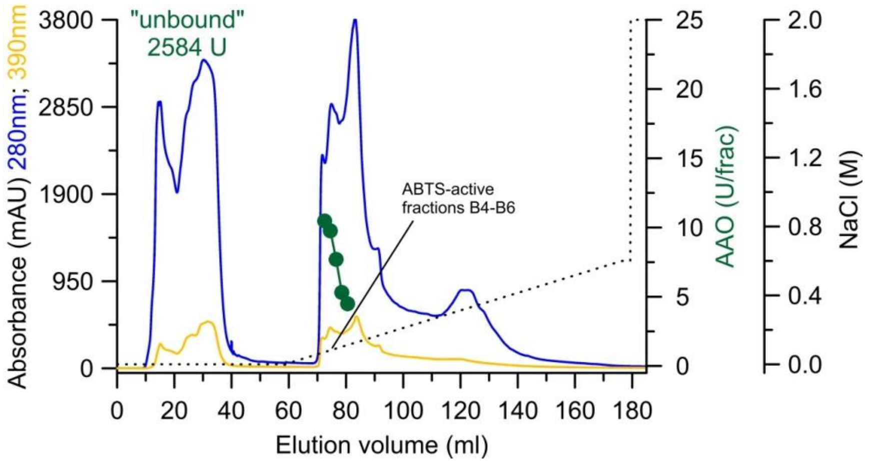

Fig. S4: Elution profile of *Post*AAO; anion exchange chromatography (MonoQ® 10/100) after applying 2,800 U *Post*AAO.

Only traces of *Post*AAO bound, probably due to residual salts, but the purification step was nevertheless successful (compare purification table S1). *Post*Lac: ABTS oxidizing fractions were pooled (B4-B6), color: bluegreen; “unbound fraction” was concentrated with viva spins: 10 kDa cut-off.

Size exclusion chromatography:
Sample preparation: dilution with SEC buffer and concentration with viva spins (10 kDa cut-off)
Column: **Sephadex®75**; 26 mm x 600 mm, GE Healthcare
Buffer: 50 Na acetate, 100 mM NaCl, pH 6.8
Flow: 2.5 mL min^{-1}, loading with a sample loop; fraction size 2 mL
around 2,500 U *Post*AAO were applied in two runs → recovery 2,000 U

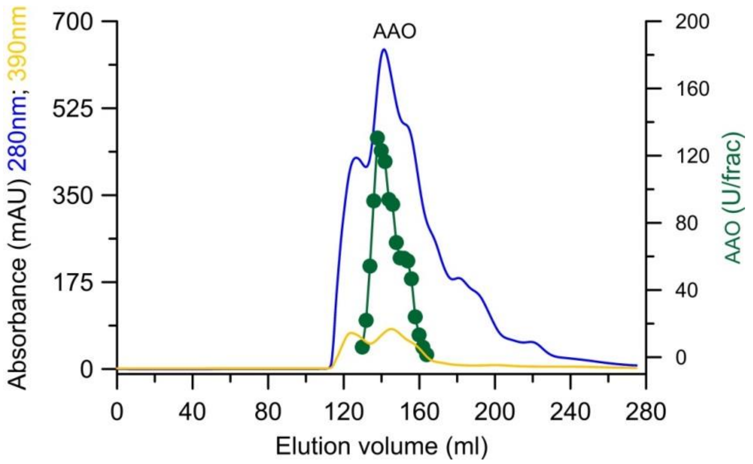

Fig. S5: Elution profile (size exclusion chromatography = SEC) on a Sephadex®75 (26/600) column after loading 1,250 U *Post*AAO.

Ion-exchange chromatography:
Sample preparation: dilution & purging with 10 mM Na acetate (pH 6.0) with viva spins (10 kDa cut-off)
Column: **MonoQ®**, 5 mm x 5 mm, GE Healthcare
Loading buffer: 10 Na acetate, pH 6.0
Elution buffer: 10 Na acetate pH 6.0 + 1 M NaCl
Flow: 2 mL min^{-1}, loading with sample pump; washing with 2.0 CV; 30% B within 35 CV; fraction size 0.8 mL; around 2,500 U *Post*AAO were applied in four runs → recovery 1,600 U

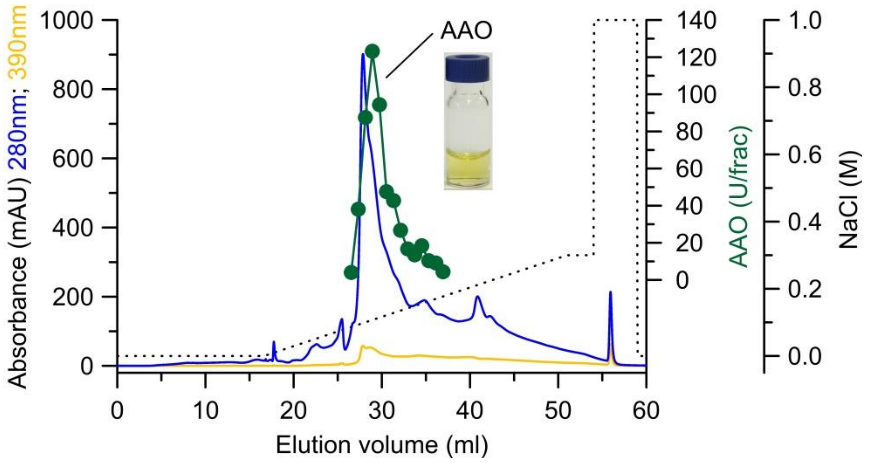

Fig. S6: Elution profile of *Post*AAO (600 U); anion exchange chromatography on MonoQ® (5/50).

Protein purification results:
Protein content: Bradford Assay with Roti®-Nanoquant, Carl Roth; 200 µL Roti®-Nanoquant + 50 µL sample in 96-well plates
Activity of aryl alcohol oxidase (AAO, E.C. 1.1.3.7): oxidation of veratryl alcohol to veratraldehyde at pH 5,5; 310 nm (ε = 9300 $M^{-1}cm^{-1}$); 50 mM Na citrate; 5 mM veratryl alcohol

Tab. S1: Purification of AAO from *Pleurotus ostreatus*

Purification step	Total activity [U]	Total protein [mg]	Specific activity [U mg^{-1}]	Purification (-fold)	Yield (%)	Volume activity (U mL^{-1})	Protein conc. (mg mL^{-1})	Volume [mL]
**Ultrafiltrate**	2,710	615	4,4	1	(100)	3.3	0.75	820
**Q-sepharose®**	2,849	245.2	11.6	3	100	142.5	12.26	20
**MonoQ® (10/100)**	2,584	138.27	18.7	4	95	172.2	9.22	15
**Sephadex® 75**	1,785	47.92	37.2	8	66	223.1	5.99	8
**MonoQ® (5/50)**	1,662	20.63	80.6	18	61	302.3	3.75	5.5

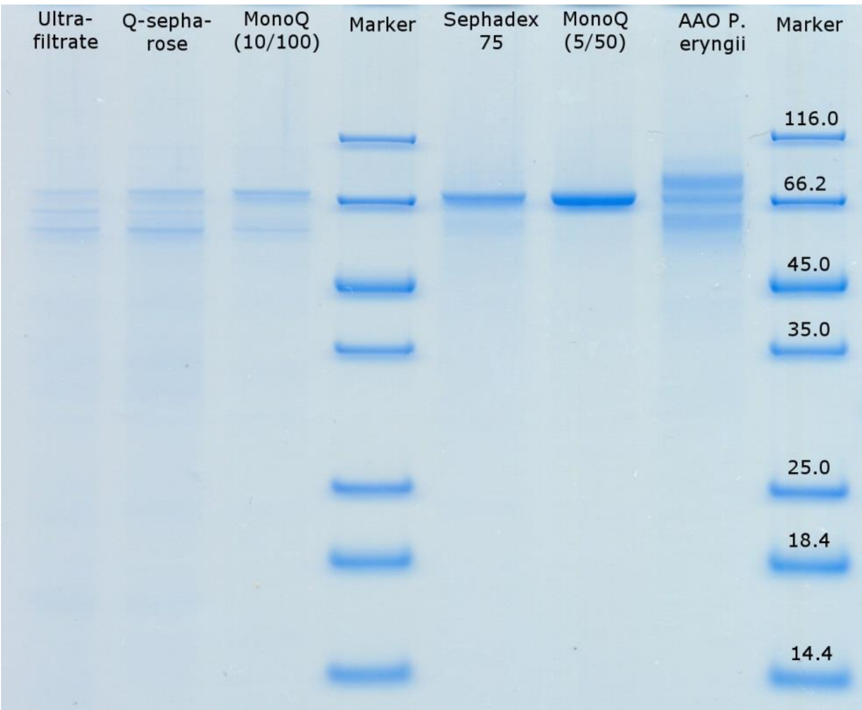

Fig. S7: SDS-PAGE of the different purification steps of AAO from *Pleurotus ostreatus* and purified AAO from *Pleurotus eryngii*; marker: Unstained Protein Molecular Weight Marker, Thermo Scientific; NuPAGE® Novex® Bis-Tris Mini-Gels 12%, Invitrogen; Conditions: 45 min, 200 V, 120 mA; staining with Novex® Colloidal Blue Stain Kit, Invitrogen

2.1 Analytical method

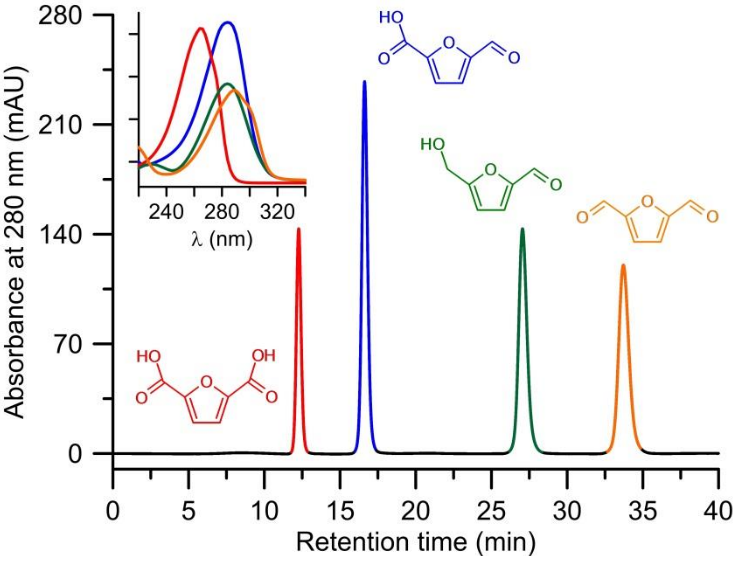

Fig. S 8: HPLC elution profile of a mixture containing HMF (green), DFF (orange), FFCA (blue) and FDCA (red); the inset displays the corresponding UV-spectra.

3.1 pH optimum of DFF oxidation

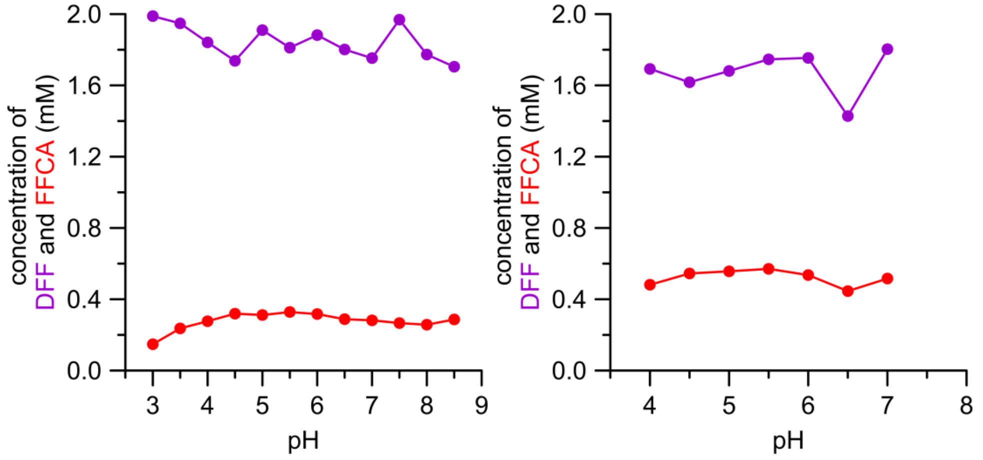

Fig. S9: pH dependencies of *Pery*AAO (A) and *Post*AAO (B) for DFF conversion. Violet curve – DFF concentration; red curve - FFCA concentration

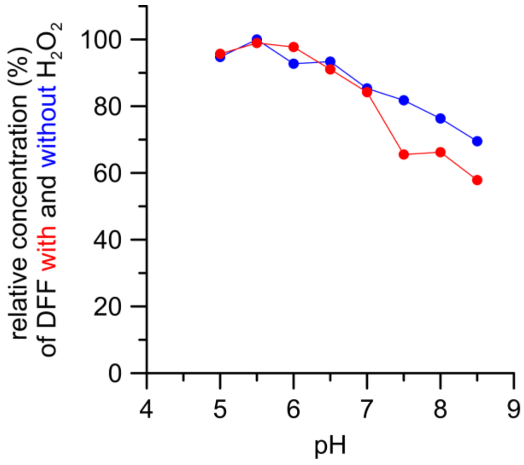

Fig. S10: pH dependency of DFF conversion catalyzed by GAO. Blue curve – relative concentration of DFF with H_2O_2; red curve – relative concentration of DFF without H_2O_2

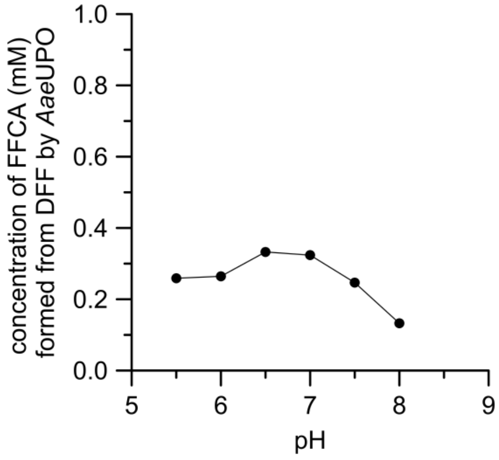

Fig. S11: pH dependent DFF oxidation catalyzed by *Aae*UPO. Black curve – DFF concentration

3.2 Calculation of apparent kinetic constants

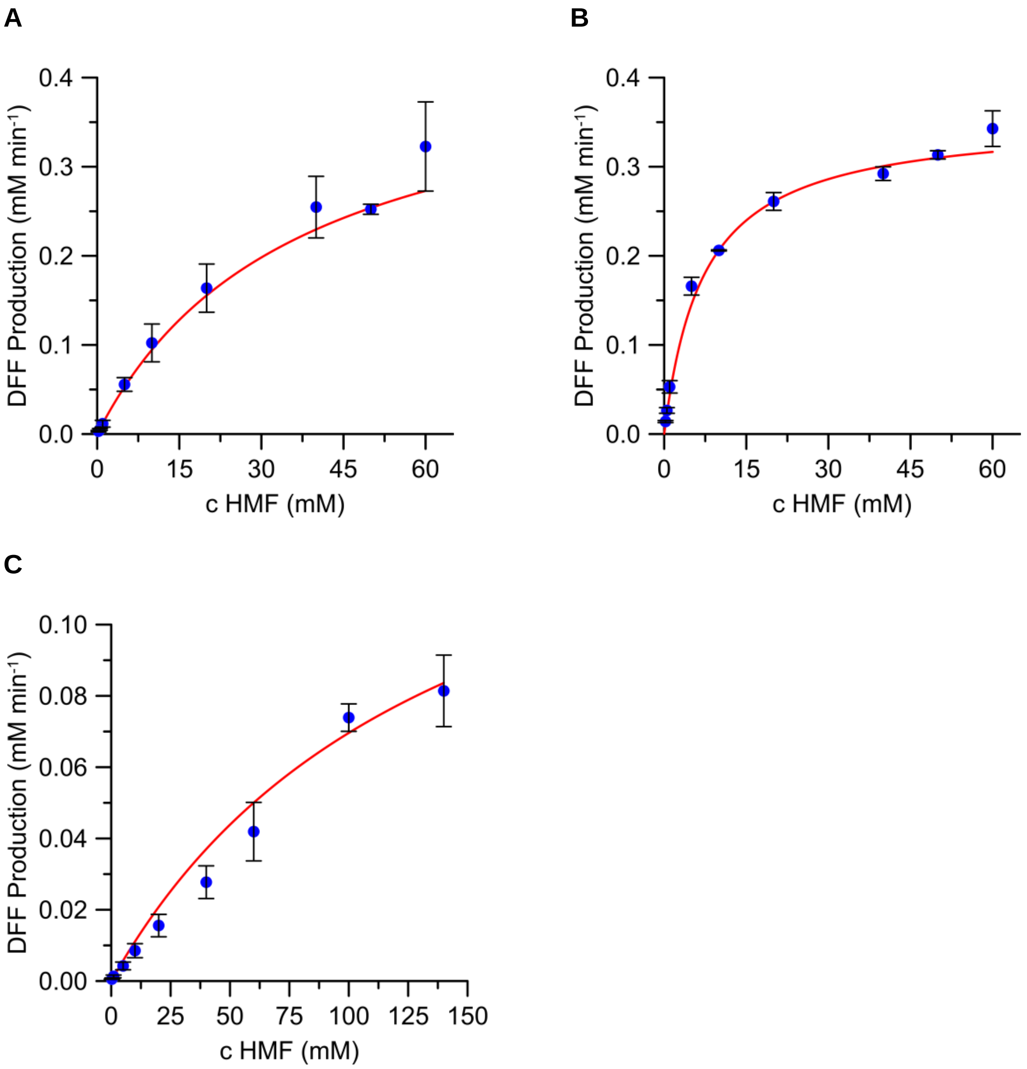

Fig. S12: Michaelis-Menten plots and their Lineweaver-Burk derivatives for the formation of DFF (from HMF) by *Pery*AAO (A), *Post*AAO (B) and GAO (C)

Setup:

pH: 2 mM HMF, 0.6 mg mL^{-1} GAO, KP_i 30 mM, 1 µL NOVOZYMES Catalase (left), shaking for 2 h in 1.5-mL Eppendorf tubes

MM-Kin.: at pH 6.0, 4 µM GAO, stopped with Na azide (1 mM)

pH: 2 mM HMF, 60 µg mg mL AAO, KP_i 30 mM, 1 µL , shaking for 2 h in 1.5-mL Eppendorf-tubes

MM-Kin.: at pH 6.0, 2 µM AAO, stopped with Na azide (1 mM)

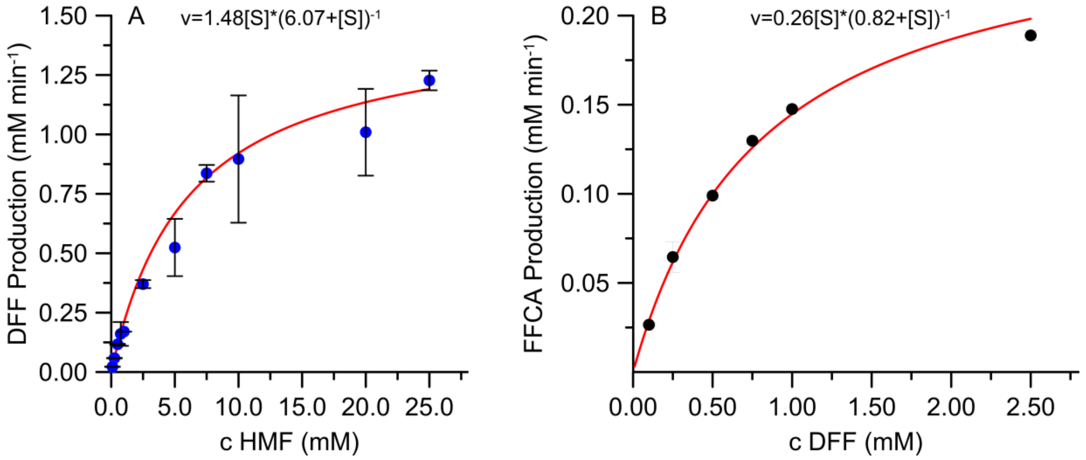

Fig. S13: Michaelis-Menten plots and their Lineweaver-Burk derivatives for the formation of DFF (A) and FFCA (B) by *Aae*UPO.

Setup MM-Kin.: at pH 6.0, 0.111 µM *Aae*UPO, stopped with Na azide (1 mM)

3.3 AAO-dependent FFCA-oxidation with regard to varying H_2O_2 concentration

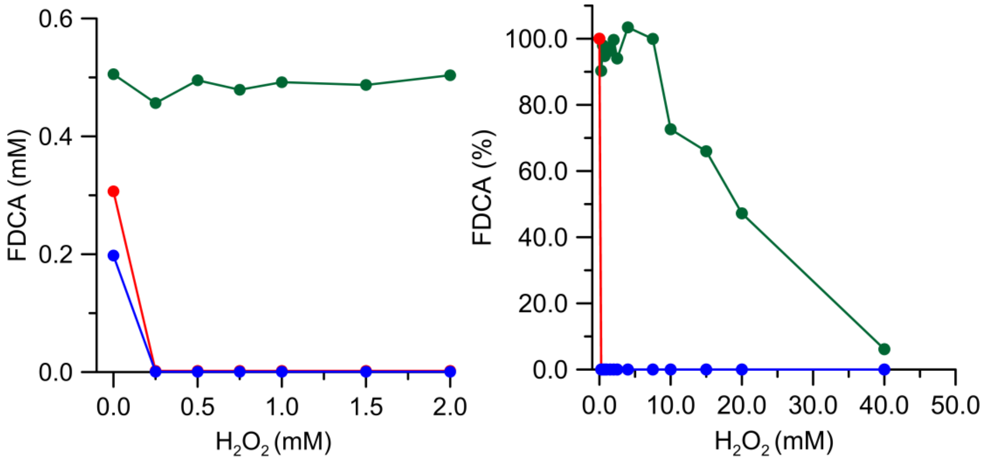

Fig. S14: FDCA formation catalyzed by *Badu*AAO (red), *Post*AAO (blue) and *Pery*AAO (green) with regard to different concentrations of H_2O_2 supplied (left) and relative FDCA production of *Badu*AAO (red), *Pos*AAO (blue) and *Pery*AAO (green) (right).

Tab. S2: FDCA formation catalyzed by selected AAOs with regard to pH

	FDCA [mM]		
	pH 6	pH 7	pH 7.5
*Pery*AAO	0.008±0.00	1.706±0.21	0.903±0.09
*Post*AAO	0.004±0.00	0.012±0.00	0.004±0.00
*Badu*AAO	0.067±0.01	0.605±0.11	1.583±0.23
